# Estimating Asian Contribution to the Brazilian Population: A New Application of a Validated Set of 61 Ancestry Informative Markers

**DOI:** 10.1534/g3.118.200650

**Published:** 2018-09-05

**Authors:** Roberta B. Andrade, Marcos A. T. Amador, Giovanna C. Cavalcante, Luciana P. C. Leitão, Marianne R. Fernandes, Antônio A. C. Modesto, Fabiano C. Moreira, André S. Khayat, Paulo P. Assumpção, Ândrea Ribeiro-dos-Santos, Sidney Santos, Ney P. C. Santos

**Affiliations:** * Núcleo de Pesquisas em Oncologia, Universidade Federal do Pará, Belém, Pará, Brazil, 66073-005; † Laboratório de Genética Humana e Médica, Universidade Federal do Pará, Belém, Pará, Brazil, 66075-970

**Keywords:** Ancestry-informative markers, Insertion-deletion polymorphisms, Admixture, Population substructure, Asian

## Abstract

Estimates of different ancestral proportions in admixed populations are very important in population genetics studies, especially for the detection of population substructure effects in studies of case-control associations. Brazil is one of the most heterogeneous countries in the world, both from a socio-cultural and a genetic point of view. In this work, we investigated a previously developed set of 61 ancestry informative markers (AIM), aiming to estimate the proportions of four different ancestral groups (African, European, Native American and Asian) in Brazilian populations. To the best of our knowledge, this is the first study to use a set of AIM to investigate the genetic contribution of all four main parental populations to the Brazilian population, including Asian contribution. All selected markers were genotyped through multiplex PCR and capillary electrophoresis. The set was able to successfully differentiate the four ancestral populations (represented by 939 individuals) and identify their genetic contributions to the Brazilian population. In addition, it was used to estimate individual interethnic admixture of 1050 individuals from the Southeast region of Brazil and it showed that these individuals present a higher European ancestry contribution, followed by African, Asian and Native American ancestry contributions. Therefore, the 61 AIM set has proved to be a valuable tool to estimate individual and global ancestry proportions in populations mainly formed by these four groups. Our findings highlight the importance of using sets of AIM to evaluate population substructure in studies carried in admixed populations, in order to avoid misinterpretation of results.

Studies of genetic association are frequently performed in groups of non-related individuals in one or more populations to identify susceptibility *loci* regarding complex human traits (Santos *et al.* 2010). However, allelic frequencies vary between ethnically different populations and, if this fact is neglected in studies carried in admixed populations, it could lead to misinterpretation of results (Collins-Schramm *et al.* 2002). Therefore, it is important to estimate the genetic contributions in such populations, as well as controlling the substructure effects in these types of studies.

Brazil is one of the most heterogeneous countries in the world, both from a socio-cultural and a genetic point of view. The history of this country is marked by continental migrations, which influenced in a definitive manner the process of admixture of the Brazilian population. In the first half of the 16^th^ century, Europeans (mainly Portuguese) started an immigration process to the large territory that was inhabited by Native Americans (also known as Amerindians) and later would become Brazil (Cunha 1995). In the second half of the 16^th^ century, the process of slave trade from Africa started and it continued until 1850 (Curtin 1969).

The immigration process of Asians to Brazil started in the beginning of the 20^th^ century, with the arrival of the first Japanese immigrants in the city of Santos (located in São Paulo state, in the Southeastern Brazil), brought by the Kasato-Maru ship (Takenaka 2003). Currently, Brazil is home of approximately 1.5 million Japanese, becoming the largest Japanese community outside Japan (Suarez-Kurtz 2010). It shows the importance of this parental group to the formation of the Brazilian population. However, most studies still only consider European, Native Americans and African as the main contributors to the admixed Brazilian population and this could lead to misinterpretation of results.

Therefore, when carrying association studies in the Brazilian population, it is important to consider that: i) the current Brazilian population was mainly formed by the admixture of four continental populations (Native Americans, Europeans, Africans and Asians); and ii) association studies carried in admixed populations should be carefully analyzed to avoid misinterpretation due to population substructure, which can be done through ancestry informative markers (AIM). These markers, also known as population-specific markers, are genetic markers with great variation of allelic frequency between different continental populations (*i.e.*, a certain allele can range from exclusive absence to exclusive presence in different populations) (Parra *et al.* 2003).

Estimates of genetic ancestry proportions in admixed populations are not only fundamental to control the effect of population substructure in association studies, but they can also be useful in other types of investigations, being considered more accurate than physical traits (Ramos *et al.* 2016). AIM sets have been developed and used to answer questions related to epidemiology, forensic anthropology, pharmacogenetics and population genetics (Parra *et al.* 1998).

The aim of this study was to evaluate how efficient a 61 INDEL-type AIM set would be to estimate the Asian contribution (represented by Japanese) in a sample of individuals from the São Paulo population.

## Material And Methods

### Investigated Samples

This study included 939 samples to represent the parental groups that contributed to the formation of the Brazilian population and 1050 individuals from the admixed population of São Paulo state, Brazil. The samples considered as parental groups included: 200 Sub-Saharan African individuals, from Angola, Mozambique, Zaire, Cameroon and the Ivory Coast; 290 European individuals, mainly from Portugal and Spain; 246 Native American individuals from tribes in the Brazilian Amazon (Tiriyó, Waiãpi, Zoé, Urubu-Kaapor, Awa-Guajá, Parakanã, Wai Wai, Gavião, Zoró); and 203 Japanese individuals residing in the North region of Brazil that were either: i) immigrants born in Japan; or ii) Brazilian individuals with Japanese parents or grandparents. With the exception of the Japanese group, all samples were collected in the origin country and have been previously described by (Santos *et al.* 2010; Ramos *et al.* 2016).

Furthermore, to validate the AIM set usage for estimating Asian ancestry, we employed it in the analyses of 1050 individuals from São Paulo state, in Southeastern Brazil. This population was formed by the admixture of European (higher contribution), African and Native American populations, as well as, more recently, by a significant amount of Japanese individuals. Therefore, the São Paulo population is suitable to be analyzed in this study.

All participants have authorized the collection of their biological samples by signing a consent form and the ethical aspects of this study have been approved by the Ethics Committee (Santos *et al.* 2010).

### DNA Extraction

DNA was extracted from peripheral blood with Biopur Kit Mini Spin Plus (Biopur, Brazil). DNA quantification was performed by a spectrophotometer (Nano Drop 1000, Thermo Fisher Scientific, Wilmington, DE, USA). Further, the samples were diluted to a 10 ηg/μL concentration and used for the multiplex PCR.

### Selection of Markers

In this study, we used a set of 61 INDEL-type markers that was described by Santos *et al.* and Ramos *et al.* It is noteworthy that, as far as we know, this is the AIM panel with the highest number of INDEL markers to be applied in the Brazilian population to date.

### Multiplex PCR and Fragment Analysis

Genotyping of the 61-AIM set was performed by Multiplex PCR (two runs), followed by capillary electrophoresis with fragment analysis, as described by Santos *et al.* and Ramos *et al.* Technical features of the used primers can be found in these published works. For the multiplex amplifications, we used the following protocol for each sample: 5.0 μL of the QIAGEN Multiplex PCR kit (QIAGEN, Germany), 1.0 μL of Q-solution, 1.0 μL of Primer Mix, 2.0 μL of water and 1.0 μL of DNA (concentration of 10ng/μL). PCR was performed in the ABI Veriti thermocycler (Life Technologies, Foster City, CA, USA) with the following program: 95° for 15 min; 35 cycles 94° for 45 sec, 60° for 90 sec and 72° for 60 sec; 70° for 30 min.

For the capillary electrophoresis and fragment analysis, we used the following protocol for each sample: 1.0 μL of the PCR product to each 8.5 μL of deionized formamide HI-FI (Life Technologies) and 0.5 μL of GeneScan 500 LIZ pattern size (Life Technologies). Separation of DNA fragments was performed using ABI PRISM 3130 Genetic Analyzer and GeneMapper ID v3.2 software (Life Technologies) was used for peak reading.

### Statistical Analyses

We used Arlequin v.3.5 software (Excoffier and Lischer 2010) to verify Fixation index (FST) and to analyze Hardy-Weinberg Equilibrium (HWE) and allelic frequency of the markers in the studied samples. Analyses and construction of graphs with the individual schematic representation of individual admixture estimates and Discriminant Analysis of Principal Components (DAPC) were performed in the R environment, using *adegenet* package (R core team 2018; Jombart and Ahmed 2011). For all of the other ancestry analyses, we used the Structure v2.3.4 software (Pritchard *et al.* 2000; Falush *et al.* 2003; Falush *et al.* 2007; Hubisz *et al.* 2009). P-value ≤ 0.05 was considered statistically significant.

### Data and reagent availability

The authors affirm that all data necessary for confirming the conclusions of the article are present within the article, figures, and tables. Supplemental material available at Figshare: https://doi.org/10.25387/g3.7040102.

## Results

Table 1 shows allelic frequencies data of all investigated markers in the four parental populations and in the admixed population of São Paulo. There was no deviation from HWE in the investigated populations regarding genotype distributions of the markers.

**Table 1:** Short allele (or deletion allele) frequencies of the 61 markers in african (AFR), european (EUR), native american (NAM), asian (ASN) populations and in the admixed population of são paulo (SP)

Markers	ASN	EUR	NAM	AFR	SP
**rs140762**	0.56	0.4	0	0.96	0.45
**rs2308144**	0.7	0.3	0.91	0.18	0.32
**rs1160850**	0.07	0.7	0.4	0.64	0.64
**rs16710**	0.58	0.67	1	0.16	0.62
**rs2307644**	0.13	0.53	0.01	0.07	0.34
**rs1610941**	0.6	0.84	0.27	0.42	0.73
**rs140847**	0.7	0.18	0.51	0.23	0.27
**rs2307828**	0.82	0.22	0.66	0.92	0.36
**rs2067259**	0	0.24	0	0.51	0.25
**rs16383**	0.91	0.8	0.01	0.17	0.64
**rs1610864**	0.37	0.79	0.3	0.74	0.71
**rs2067128**	0.48	0.48	0.24	0.14	0.44
**rs2307799**	0.53	0.4	0.92	0.07	0.38
**rs2307582**	0.32	0.14	0.71	0.17	0.21
**rs2307981**	0.4	0.23	0.02	0.63	0.3
**rs2307976**	0.55	0.73	0.44	0.32	0.63
**rs2307666**	0.17	0.64	0	0.14	0.46
**rs1610875**	0.7	0.87	0.94	0.26	0.75
**rs4183**	0.83	0.31	0.42	0.73	0.43
**rs2307587**	0.64	0.95	0.81	0.66	0.88
**rs16653**	0.7	0.85	0.34	0.71	0.79
**rs2308261**	0.8	0.39	0.69	0.34	0.41
**rs2067186**	0.74	0.39	0.27	0.31	0.41
**rs25574**	0.67	0.05	0.45	0.08	0.1
**rs1611095**	0.4	0.79	0.27	0.3	0.65
**rs1611070**	0.89	0.3	0.72	0.11	0.36
**rs2067270**	0.37	0.25	0.83	0.15	0.26
**rs2308203**	0.82	0.25	0.81	0.18	0.68
**rs25549**	0.58	0.48	0.44	0.93	0.55
**rs2307922**	0.78	0.65	0.63	0.2	0.6
**rs2308205**	0.54	0.19	0.9	0.06	0.27
**rs2307553**	0.55	0.7	1	0.12	0.6
**rs2308115**	0.97	0.22	0.53	0.52	0.4
**rs2307880**	0.22	0.4	0.17	0.48	0.4
**rs140765**	0.77	0.31	0.85	0	0.31
**rs1160871**	0.17	0.25	0.81	0.62	0.25
**rs16712**	0.52	0.7	0.43	0.63	0.64
**rs1610902**	0.04	0.24	0.01	0.74	0.3
**rs16432**	0.89	0.2	0.94	0.22	0.28
**rs2307659**	0.31	0.17	0.35	0.8	0.28
**rs16635**	0.58	0.54	0.04	0.53	0.48
**rs16388**	0.63	0.47	0.99	0.8	0.57
**rs2067263**	0.92	0.33	0.2	0.89	0.43
**rs2307912**	0.73	0.87	0.66	0.34	0.8
**rs2307554**	0.76	0.87	0.84	0.41	0.77
**rs16343**	0.93	0.53	0.73	0.08	0.54
**rs1160894**	0.82	0.6	0.88	0.6	0.58
**rs25546**	0.53	0.3	0.57	0.88	0.41
**rs2067353**	0.62	0.86	0.3	0.1	0.73
**rs140864**	0.7	0	0.71	0.1	0.09
**rs16416**	0.22	0.61	0.59	0.2	0.52
**rs2067141**	0.23	0.71	0.56	0.2	0.53
**rs140783**	0.39	0.77	0.62	0.26	0.64
**rs140770**	0.7	0.32	0	0.12	0.23
**rs1610996**	0.05	0	0.13	0.02	0.01
**rs1160876**	0.96	0.68	0.85	0.28	0.66
**rs139049210**	0.35	0.58	0.4	0.09	0.49
**rs16654**	0.65	0.49	0.12	0.04	0.43
**rs140857**	0.22	0.45	0.06	0.1	0.38
**rs2067271**	0.9	0.42	0.78	0.28	0.44
**rs1611106**	0.46	0.83	0.7	0.38	0.75

These data were used to estimate delta values (δ), which are positive values that correspond to the frequency differences between two populations. The delta values of the comparison of the four parental populations are presented in Table 2.

**Table 2:** Observed delta values (Δ) in the different comparisons between the four parental populations

Markers	ASN/EUR	ASN/NAM	ASN/AFR	EUR/NAM	EUR/AFR	NAM/AFR
**rs140762**	0.16	0.56	0.4	0.4	0.56	0.96
**rs2308144**	0.4	0.21	0.52	0.61	0.12	0.73
**rs1160850**	0.63	0.33	0.57	0.3	0.06	0.24
**rs16710**	0.09	0.42	0.42	0.33	0.51	0.84
**rs2307644**	0.4	0.12	0.06	0.52	0.46	0.06
**rs1610941**	0.24	0.33	0.18	0.57	0.42	0.15
**rs140847**	0.52	0.19	0.47	0.33	0.05	0.28
**rs2307828**	0.6	0.16	0.1	0.44	0.7	0.26
**rs2067259**	0.24	0	0.51	0.24	0.27	0.51
**rs16383**	0.11	0.9	0.74	0.79	0.63	0.16
**rs1610864**	0.42	0.07	0.37	0.49	0.05	0.44
**rs2067128**	0	0.24	0.34	0.24	0.34	0.1
**rs2307799**	0.13	0.39	0.46	0.52	0.33	0.85
**rs2307582**	0.18	0.39	0.15	0.57	0.03	0.54
**rs2307981**	0.17	0.38	0.23	0.21	0.4	0.61
**rs2307976**	0.18	0.11	0.23	0.29	0.41	0.12
**rs2307666**	0.47	0.17	0.03	0.64	0.5	0.14
**rs1610875**	0.17	0.24	0.44	0.07	0.61	0.68
**rs4183**	0.52	0.41	0.1	0.11	0.42	0.31
**rs2307587**	0.31	0.17	0.02	0.14	0.29	0.15
**rs16653**	0.15	0.36	0.01	0.51	0.14	0.37
**rs2308261**	0.41	0.11	0.46	0.3	0.05	0.35
**rs2067186**	0.35	0.47	0.43	0.12	0.08	0.04
**rs25574**	0.62	0.22	0.59	0.4	0.03	0.37
**rs1611095**	0.39	0.13	0.1	0.52	0.49	0.03
**rs1611070**	0.59	0.17	0.78	0.42	0.19	0.61
**rs2067270**	0.12	0.46	0.22	0.58	0.1	0.68
**rs2308203**	0.57	0.01	0.64	0.56	0.07	0.63
**rs25549**	0.1	0.14	0.35	0.04	0.45	0.49
**rs2307922**	0.13	0.15	0.58	0.02	0.45	0.43
**rs2308205**	0.35	0.36	0.48	0.71	0.13	0.84
**rs2307553**	0.15	0.45	0.43	0.3	0.58	0.88
**rs2308115**	0.75	0.44	0.45	0.31	0.3	0.01
**rs2307880**	0.18	0.05	0.26	0.23	0.08	0.31
**rs140765**	0.46	0.08	0.77	0.54	0.31	0.85
**rs1160871**	0.08	0.64	0.45	0.56	0.37	0.19
**rs16712**	0.18	0.09	0.11	0.27	0.07	0.2
**rs1610902**	0.2	0.03	0.7	0.23	0.5	0.73
**rs16432**	0.69	0.05	0.67	0.74	0.02	0.72
**rs2307659**	0.14	0.04	0.49	0.18	0.63	0.45
**rs16635**	0.04	0.54	0.05	0.5	0.01	0.49
**rs16388**	0.16	0.36	0.17	0.52	0.33	0.19
**rs2067263**	0.59	0.72	0.03	0.13	0.56	0.69
**rs2307912**	0.14	0.07	0.39	0.21	0.53	0.32
**rs2307554**	0.11	0.08	0.35	0.03	0.46	0.43
**rs16343**	0.4	0.2	0.85	0.2	0.45	0.65
**rs1160894**	0.22	0.06	0.22	0.28	0	0.28
**rs25546**	0.23	0.04	0.35	0.27	0.58	0.31
**rs2067353**	0.24	0.32	0.52	0.56	0.76	0.2
**rs140864**	0.7	0.01	0.6	0.71	0.1	0.61
**rs16416**	0.39	0.37	0.02	0.02	0.41	0.39
**rs2067141**	0.48	0.33	0.03	0.15	0.51	0.36
**rs140783**	0.38	0.23	0.13	0.15	0.51	0.36
**rs140770**	0.31	0.7	0.11	0.32	0.2	0.12
**rs1610996**	0.05	0.08	0.03	0.13	0.02	0.11
**rs1160876**	0.28	0.11	0.68	0.17	0.4	0.57
**rs139049210**	0.23	0.05	0.26	0.18	0.49	0.31
**rs16654**	0.16	0.53	0.61	0.37	0.45	0.08
**rs140857**	0.23	0.16	0.12	0.39	0.35	0.04
**rs2067271**	0.48	0.12	0.62	0.36	0.14	0.5
**rs1611106**	0.37	0.24	0.08	0.13	0.45	0.32
**MEAN**	**0.31**	**0.24**	**0.35**	**0.35**	**0.33**	**0.4**

The mean δ was higher in the comparison between NAM and AFR (0.40), and the lower mean δ was observed between ASN and NAM (0.24), which suggests a greater proximity between the NAM and ASN, corroborating the results by (Ribeiro-dos-Santos *et al.* 2013). The mean δ of the comparisons ASN/EUR and ASN/AFR do not differ much from the observed means between the other previously investigated continental populations: EUR and NAM (0.35), EUR and AFR (0.33) and NAM and AFR (0.40). These values seem to be within the expected and they corroborate the work of Santos *et al.*

Considering that the δ results indicate that this AIM panel is suitable to estimate interethnic admixture with Asian in individuals from admixed populations, we performed additional analyses. We quantified the level of standard error (SE) using data obtained in analysis performed with Structure v.2.3.4. software. As described by Halder *et al.* [18], SE represents the bias caused by the nature of allele frequency distributions, not the bias that could be generated by the process of sample selection, and it can be defined as population SE (total ancestry from the noncontributing populations to individuals) or ancestry SE (total contribution from one noncontributing population to other populations). Table 3 shows the results from these analyses. In the four parental populations, the estimate of interethnic admixture showed more than 99% similarity within the expected group, which establishes the mean population SE with less than 1%.

**Table 3:** Quantification of statistical bias level introduced in interethnic admixture estimates

	EUR	NAM	AFR	ASN	Population SE
EUR	0.998	0	0.001	0.001	0.002
NAM	0	0.998	0	0.002	0.002
AFR	0.001	0	0.999	0	0.001
ASN	0.001	0.002	0	0.997	0.003
Ancestry SE	0.002	0.002	0.001	0.003	

**Figure 1:**
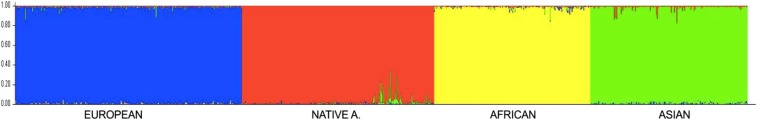
Schematic representation of the interethnic admixture estimates in the four continental populations, using Structure software for k = 4. Barplot: each vertical line represents an individual and the proportion of admixture of European (blue), Native American (red), African (yellow) and Asian (green) populations.

The measure of ancestral SE was also very low (less than 1%), which means that none of the parental populations could have contributed with more than 1% in the formation of the other three populations. It suggests that the panel of 61 AIM could be employed in the estimates of individual interethnic admixture involving the Asian ancestral population. Figure 1 presents the estimates of genetic contribution for each individual included in the study and it shows that the panel of 61 AIM successfully differentiates European, African, Native American and Asian populations.

**Figure 2:**
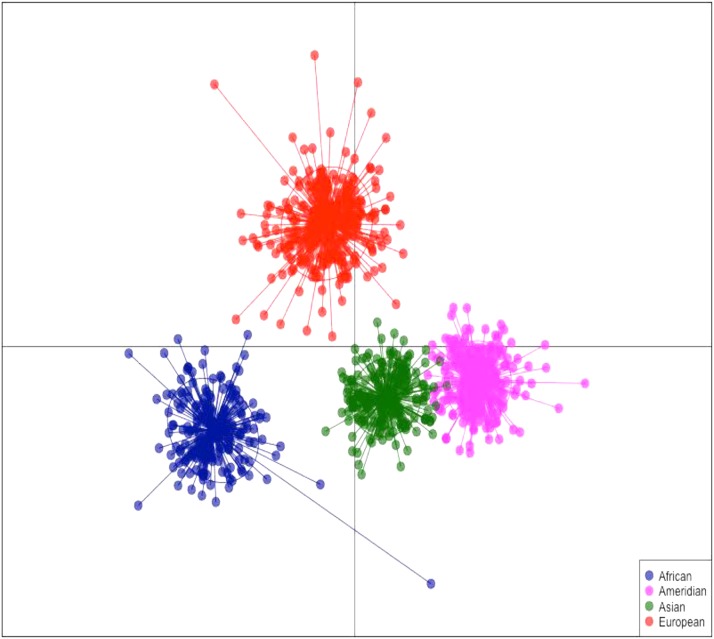
DAPC analysis in the four populations: Africans form the blue cluster, Europeans form the red cluster, Native Americans form the lilac cluster and Asians form the green cluster.

Furthermore, we performed the DAPC analysis in the four parental populations, which generated four distinct clusters (Figure 2). It is noteworthy that the populations described as more genetically similar (Native Americans and Asians) are still clearly separated.

**Figure 3:**
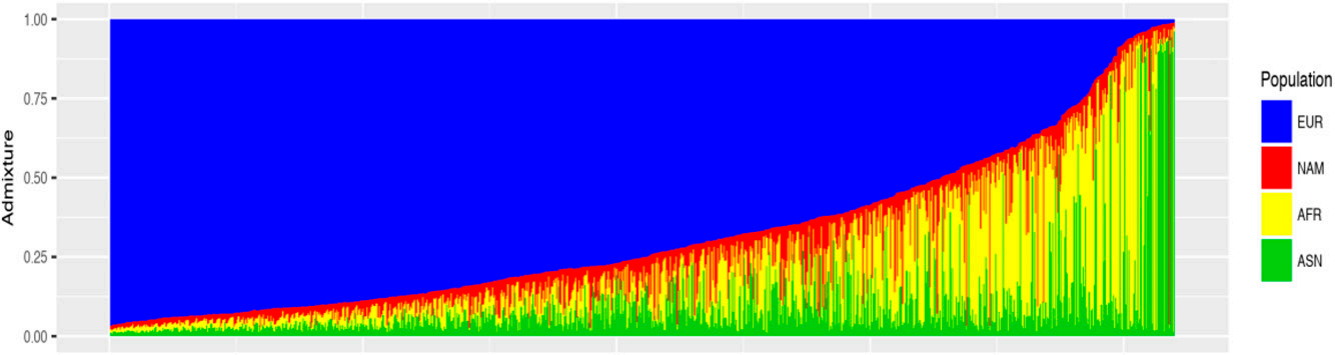
Schematic representation of the individual estimates in Brazilian admixed populations (São Paulo). Bar plot: each vertical line represents one individual and the correspondent European (blue), Native American (red), African (yellow) and Asian (green) contributions.

For the investigated markers, the FST values show a difference of 0.32 between European and Native American; 0.32 between European and African; 0.26 between European and Asian; 0.43 between Native American and African; 0.23 between Native American and Asian; and 0.35 between African and Asian. Considering the obtained values, the FST results indicate a great degree of differentiation between Native American and Asian populations, and an even greater degree in the analysis between the other populations (all with P-value < 0.05). To further investigate this finding, we applied the 61-AIM set to estimate interethnic admixture in samples from 1050 individuals from the São Paulo population that have reported Asian ancestry. Then, we performed analyses considering three and four parental populations (EUR/AFR/NAM; EUR/AFR/ASN and EUR/AFR/NAM/ASN) (Table 4). European contribution presented the higher contribution in the São Paulo sample, with similar percentage, regardless of the carried test: i) four ancestral populations (67.5%), ii) three ancestral populations without ASN (68.4%) and iii) three ancestral populations without NAM (67.8%). There is also a small variation in the AFR contribution between the three analyses: 16.1% when considering the four ancestral populations, 19.6% when considering three ancestral populations without ASN and 18.2% when considering the three ancestral populations without NAM. Schematic representation of the individual admixture estimates in Brazilian admixed populations (São Paulo) is presented in Figure 3.

**Table 4:** Percentage (%) of global interethnic admixture estimates in the studied são paulo population

	EUR/AFR/NAM	EUR/AFR/ASN	EUR/AFR/NAM/ASN
EUR	68.4	67.8	67.5
AFR	19.6	18.2	16.1
NAM	12	—	6.6
ASN	—	14	9.8

In addition, we present the estimates of individual interethnic admixture in Figures S1, S2 and S3. Both European and African proportions showed a great similarity in these estimates, when considering three or four ancestral populations.

Here, we highlight that the NAM and ASN contributions show similar percentage when analyzed separately (12% and 14%, respectively), but when they are considered in the same analysis, they are notably different (6.6% and 9.8%, respectively), as shown in Table 4. These percentages suggest that the contribution of these populations, when analyzed together, is split between them.

When we analyzed the contribution from all four populations, we observed that 0.6% of the studied individuals from the Southeast region presented at least 30% of Asian contribution. From these individuals, 3% had more than 70% of Asian contribution.

## Discussion

In this study, we aimed to determine whether a 61-AIM set with reported efficiency in estimating individual interethnic admixture in three ancestral groups (European, African and Native American) (Santos *et al.* 2010; Ramos *et al.* 2016) would be able to infer Asian ancestry and measure the Asian component in Brazilian admixed populations. For this purpose, in addition to groups representing European, Native American and African ancestries, we included a population from the Northern Brazil with known Asian origin and a population from the Southeastern Brazil with admixture history of the 4 populations (EUR, AFR, ASN and NAM).

To validate the AIM panel, we employed an approach that has been previously used (Halder *et al.* 2008; Santos *et al.* 2010) and observed that this set provides reliable estimates of the admixture of the four ancestral populations that are the main contributors to the formation of the Brazilian population (Native American, African, European and Asian). This was further strengthened by the results of the DAPC analysis with the set of markers, in which all populations are visibly separated. Moreover, the obtained FST values are indicating large or very large genetic differentiation between the four populations, according to the guidelines proposed by Wright (Wright 1978) (FST < 0.05 indicates small genetic differentiation, 0.05 < FST < 0.15 indicates moderate genetic differentiation, 0.15 < FST < 0.25 indicates large differentiation and FST > 0.25 indicates very large genetic differentiation).

Corroborating previous studies (Wallace *et al.* 1985; Schurr *et al.* 1990; Horai *et al.* 1994) our results show a greater similarity between Native American and Asian populations, when compared to the other continental groups investigated here. For instance, the mean δ between Asian and Native American populations (0.244) are lower than the correspondent measures between Asian and European populations (0.307) and between Asian and African populations (0.353).

Although there is a greater proximity between Native American and Asian groups, the AIM panel provides a robust and specific estimate of ASN individual ancestry in admixed populations, successfully separating ASN population from others. This was strengthened with the obtained results of the analyses in the samples from São Paulo, in which we were able to identify 0.6% of global Asian ancestry in this admixed population from the Southeast region of Brazil. This result differs from the registered data of 1.9% of Asian contribution in this region (IBGE 2008). However, this registered data were estimated based on self-declaration criteria, which is different from measures of genomic ancestry.

In conclusion, we demonstrated that this INDEL panel not only can be used to genetically distinguish different continental populations (specifically Europeans, Africans, Native Americans and Asians), but it can also identify substructure in admixed populations. When applied to such populations, this panel allows estimates of the individual and global interethnic admixture regarding the genetic contributions of these ancestry groups. Moreover, it was able to efficiently separate Asian and Native American populations, despite their proximity when compared to the other continental populations. Therefore, we showed in this study that the 61-AIM panel is a useful tool that could be valuable in studies in Brazilian populations, which is extremely important to avoid misinterpretations of the findings in association studies. To the best of our knowledge, this is the first study to apply an AIM panel to estimate the individual contribution of these four main parental populations (European, Native American, African and Asian) in an admixed population from Brazil.
